# Third Day

**Published:** 1881-06

**Authors:** 


### THIRD DAY—THURSDAY, MAY 5, 1881.

The Association was called to order at 10 o'clock by the President, Dr. John T. Hodgen.

Prayer by Rev. Dr. Wm. E. Hatcher, of the Grace street Baptist Church.

The report of the Committee of Arrangements for the day's proceedings was read by Dr. Frank D. Cunningham, and adopted.

On motion of Dr. Gross, the rules were suspended. He then offered a resolution to so amend the By-Laws as to establish an additional Section, to be known as the Section of Dentistry, which was adopted.

ADDRESS OF DR. HUNTER MCGUIRE, Chairman of Section on Surgery, etc., on *Operative Interference in Gunshot Wounds of Peritoneum.*—Few surgeons have had greater experience in treating gunshot wounds occurring both in military and civil life, and few have appreciated more fully how unsatisfactory are the results obtained from the expectant or do-nothing plan so much in vogue. The title of the paper indicates the grounds taken by the writer in favor of operative interference, and the views embodied in the paper tend to prove the position advanced by the writer, that the patient will exchange an almost certain prospect of death for at least a good chance of recovery, and should, we think, embolden surgeons to think less of expectant treatment and more of operative interference. Statistics from the Crimean, the French and the late American civil war, show that more than nine out of every ten patients with wounds of the belly, opening into the cavity of the peritoneum, perish—no other gunshot wounds being so deadly, not even penetrating and perforating wounds of the skull. In incised, punctured and gunshot wounds of the peritoneum, the general plan of treatment has been to enjoin absolute rest, give opium to prevent peristaltic action, and encourage the formation of adhesions, in the idle hope of preventing extravasation into the peritoneal cavity. It is claimed that the wound may paralyze the muscular coat of the bowel; or in small wounds the mucous coat is everted and closes the aperture, or the part injured may not be covered with peritoneum and no extravasation take place within the peritoneal cavity, or that the serous membrane covering the intestine near the point wounded may become adherent to the omentum, to the bowel or to the abdominal wall, and the orifice in the bowel become permanently closed; and last, but very rarely, the extravasated mass may become encysted, end in abscess, and discharge itself through the neighboring skin or mucous surface. In the opinion of the writer, when we remember that the alimentary canal is never completely empty, common sense teaches us, when an opening is made in any portion of the peritoneal cavity that its contents will escape; that there will probably be less resistance to the passage of fæcal matter through the unnatural aperture than along the sides of the canal itself. Gas may first be expelled, separating peritoneal surfaces, and then the fluid or solid contents of the bowel follow. Only one or two exceptions to this rule are reported in the history of the late war between the North and South. But, besides alimentary effusion, blood, air, bile and urine may also be extravasated into the peritoneal cavity. Penetrating wounds of the belly, with fæcal effusion, are rapidly followed by general acute peritonitis; ninety per cent. die within forty-eight hours. Does peritonitis from any other cause, as a rule, kill as quickly? In spite of the assertion of Malgaigne and others, that the organs contained in the belly fill the cavity to such repletion that shot wounds of that space without visceral injury are impossible, *post-mortem* examinations and experiments upon dead bodies show that wounds of the peritoneum can be made without injury to the contained viscera. It has fallen to the lot of the writer to witness four such cases. Two occurring in civil life, and being the subjects of legal investigation, careful autopsies were made. Two were soldiers dying from peritonitis, and the autopsies showed no visceral lesion. These four cases, coming under the observation of one individual, and having their exact character shown by *post-mortem* examinations, prove that such results are not impossible, and probably not as rare as we have been led to suppose. Those rare cases of recovery from penetrating wounds of the abdomen have induced surgeons to continue the expectant plan of treatment in place of what appears, at first sight, to be a desperate surgical interference. Some of the alleged recoveries may have been wounds of a portion of the large intestine not covered by peritoneum. Recovery, with fæcal fistulæ, is not uncommon in this case; others may have been penetrating wounds without visceral injury; others again may have been parietal wounds without peritoneal penetration. In connection with the four cases of gunshot wound of the peritoneum alluded to by the writer, and in which there was no visceral injury, the total absence of shock was remarkable, and no diminution of temperature. One of them (a soldier) assured the writer that he did not know that he had been wounded until some time after he had been shot. Another (wounded in a duel) insisted that he was able to stand up and give his antagonist another fire. On the other hand, in all cases with visceral lesions the shock of injury is a prominent symptom. The presence or absence of shock seems to be a diagnostic point of no little value. If to this be added sudden meteorism, the character, extent and direction of the wound, bloody discharges from the bowels or stomach, an almost certain diagnosis by rational symptoms will be reached. In reply to the question, Why are these injuries so fatal? why, after escaping death from peritonitis, shock and hæmorrhage, peritonitis is fatal in forty-eight hours? the writer attributes death to some kind of blood poisoning connected with peritonitis, just as we often see septicæmia associated with peritonitis under other circumstances, notably after parturition and ovariotomy. He believes that the blood poisoning after gunshot wounds of the peritoneum is consequent upon the pent-up, red, sero-fibrinous exudation which traumatic peritonitis invariably produces in abundance, and that if this effusion could be drained off as soon as it is formed, septicæmia might be prevented. In lacerated wounds of the abdominal walls, with exposure of the cavity, protrusion of the contents and the introduction of foreign matter into the cavity are nothing like so mortal.

In all of these cases the nature of the wound prevents union by the first intention, and drainage of abdominal effusions is effected. In the fifty-nine cases of recovery after penetrating wounds of the large intestine, fifty-five were perforating wounds, the large aperture of exit being usually on the posterior surface of the body, dependent and facilitating drainage. In one of the four instances of recovery in simple penetrating shot wound of the large bowel, the edges of the opening in the bowel were fastened to the wound in the abdominal wall, and in this, as well as in the other three cases, fæcal fistulæ were formed. Shot wounds of the pelvis are nothing like so fatal as wounds of the peritoneum higher up. Unless accompanied by grave visceral lesion, three cases out of four of penetrating or perforating wounds of the pelvis recover. Can this fact be satisfactorily explained upon any other theory than that drainage in these wounds is almost unavoidable? Indeed, in these cases we are taught to explore the wounds with the finger, remove loose pieces of bone and foreign bodies, and keep the aperture of entrance and exit open, that free vent may be given to all inflammatory products; and if the size and position of the wound do not facilitate this, we make the opening bigger and insert a drainage tube. Spencer Wells attributes the fatality after ovariotomy to some form of pyæmic fever or some form of blood poisoning so often associated with peritonitis, and thinks the lesson taught by many successful ovariotomists of providing for the escape of inflammatory matter, of great value, and one which should be profited by, by the surgeon who treats the gunshot wounds of the peritoneum. Ovariotomists even go so far as to wash out the cavity when peritonitis exists and death from septicæmia is imminent. In many of the cases of penetrating wounds of the peritoneum, the ball passes obliquely through the abdominal wall and the aperture shuts up like a valve, or if passing directly through the parieties, the aperture of entrance contracts at once and closes. To all intents and purposes the cavity is hermetically sealed, and the missile, pieces of clothing, blood from wounded vessels, fæcal effusion, if the intestine is wounded, and inflammatory products are all hopelessly imprisoned there. Can it be wondered that such wounds are fatal? In no other gunshot wounds of cavities do we allow the wound of entrance and exit to be closed. Who would think of shutting up the opening in gunshot wound of the knee-joint? During the late war, the plan of hermetically sealing up wounds of the pleura, a structure analogous to the peritoneum, proved most disastrous. In gunshot wounds of the chest involving the serous membrane, we keep the wound patent, and if not dependent, we do not hesitate, when effusion takes place, to make a counter-opening with a knife or trocar, and sometimes to flush the cavity with detergent and antiseptic lotions. In view of these facts, the writer ventures to advocate operative interference in gun-shot penetrating wounds of the peritoneum with intestinal injury, in penetrating wounds of the peritoneum with any visceral lesion, and similar cases without visceral injury. The wounds in the abdominal walls should be enlarged, or the linea alba opened freely enough to allow a thorough inspection of the injured parts. Hæmorrhage should be arrested. If intestinal wounds exist, they should be closed with animal ligatures, trimming their edges first if they are lacerated and ragged. Blood and all other extraneous matters should be carefully removed, and then provision made for drainage. If the wound of entrance is dependent, drainage may be secured by keeping this open. If the wound is a perforating one, and the aperture of exit dependent, the patency of this should be maintained, and, if necessary, a drainage tube of glass or other material introduced. If there is no wound of exit, and the wound of entrance is not dependent, then a dependent counter-opening should be made and kept open with a drainage tube. If it is urged that the means suggested are desperate, it can be said in reply that the evil is desperate enough to justify the means.

At the close of the reading, Dr. McGuire was loudly applauded, and his paper appropriately referred.

The next business in order was the Report of the Committee on Clinical Observation and Records.

Dr. J. T. Reeve, of Wisconsin, Chairman of the Section on State Medicine, who was to have read a paper, being absent, his time was occupied by Dr. J. S. Billings, U. S. A., who read a paper on Some of the Results of the Tenth Census, as Regards Mortality Statistics.

This paper was prepared to be read before the Section on State Medicine, but was of such great interest that Dr. Billings was requested to read it at the general session.

At the meeting of the American Medical Association in Atlanta, in 1879, the Chairman of the Section on Hygiene, in his address, called attention to the opportunity presented by the coming census for obtaining more satisfactory mortality statistics for the whole country than have heretofore been collected.

A letter from the Superintendent of the Census was read, in which he requested the coöperation of the medical profession of the country, both as to general influence and technical assistance, in obtaining the records desired.

Early in the census year, forms were prepared for a small register of deaths to be kept by physicians.

Each register contained twenty-four such slips, and a copy of the register, with a stamped envelope for its return at the end of the census year, was sent to every one in the United States who was reported, by his or her postmaster, to be a physician or to be addressed as such.

The registers having been received were examined by a skilled physician, who indicated on each slip the name of the cause of death to be used in tabulation. Very few of the registers were in such a condition that they could not be used for statistical purposes, although, as a matter of course, some of the causes of death reported could only be classified as unknown. This had been foreseen, and in the address above referred to it was expressly provided that the physician should use such terms as "paralysis of heart," "apnœa," etc., as equivalent to "unknown."

This point was not, however, made quite so clear as it should have been in the registers, as will be seen by examining the foot-note of instructions, although the intent was probably understood by the great majority of physicians. The number classified as "unknown" was 4,162, out of a total of 166,896, being about 25 per 1,000.

The number of *post-mortems* made in this number of deaths was 3,755, or 20.7 per 1,000.

When the examination and checking of these registers were completed, they were taken apart, and, as each leaf furnished the record of a single case, they could then be used as cards, and readily assorted and classified in various ways. The first classification was into groups by counties. Those coming from the large cities were then set aside, and the remainder, representing the character of the fatal diseases in rural districts and small towns, were so compiled as to show, by age, sex, and color, the number of deaths from each cause.

The units of area or locality used in this compilation were neither the State nor the county, but groups of counties in each State, selected according to altitude and topographical characteristics by the geographer of the census. Each State contained from two to six such groups, and thus we can group the prevailing causes of death on the sea coast on the table lands, among the hills, etc.

As the number of deaths returned by physicians upon these registers has no definite relation to the actual number of deaths which occurred in any given locality, and still less to the actual population of that locality, these registers can only be used in this connection to show the proportional frequency of certain causes or groups of causes of death to the whole number of deaths reported in them, or to each other. They have other and important uses, as I will explain presently.

The nomenclature and nosological classification adopted are essentially those used in the last census, being that prepared by the Royal College of Physicians.

The most important differences in it from the old nomenclature of the college are the abolition of the division of general diseases into two groups—A and B—and the placing in the general diseases of the various forms of diarrhœal diseases, which before were placed under diseases of the digestive organs. These modifications have been approved by the National Board of Health, and are now under consideration by the Committee of the College, which is engaged in the revision of the nomenclature.

After the compilation above referred to had been completed, the slips were used to correct and complete the lists of deaths, prepared by the enumerators. The blanks for these lists, which are known as the mortality schedules, differ from those used in previous censuses by having columns for the nativity of the father and mother of the decedent, the length of his residence in the county, the place where the disease was contracted, if not in the county, and the name of the attending physician. They also contain two supplementary schedules—one giving the names of those dying in the place, but belonging to families living in another county or State; the second, giving names of persons belonging to families residing in the place, but who have died away from home in another county or State.

The first rough count shows that about 620,000 deaths have been returned upon these schedules. To these there will be added, from the register slips above described, about 50,000 deaths, and the records of the cities from which no enumerator's schedules are received will add about 80,000 more, making a total of about 50,000 deaths returned for the year, which, for a population of 50,000,000, give a death rate of fifteen per thousand. While it is certain that this does not include all the deaths, it is evident that it is much more complete than previous censuses—the total number of deaths for that of 1850 having been 323,098, being a mortality rate of 13 9-10 per thousand. In 1860, there were returned 394,153 deaths, being a mortality rate of 12 5-10 per thousand. Upon this last, Professor Elliott constructed life tables, assuming a deficiency in returns of deaths of 41 per cent., or, in other words, that the true death rate was a little over 18 per thousand. If this were assumed as the true death rate for the last census year, the deficiency in returns would be less than 10 per cent.

From this brief statement it will be seen that General Walker is to be congratulated upon the improvement which has been effected in the Tenth Census in regard to the completeness of the mortality statistics, and also that the medical profession of the country has contributed largely to the securing of this relative completeness.

In order to obtain as much information as possible with regard to the amount and distribution of the actual deficiencies in the mortality lists, the entire registers of deaths of two States and several large cities for the census year have been copied, and will be compared with the mortality schedules furnished by the Census Enumerators from the same localities. The results of this comparison will probably be interesting and valuable.

An attempt has been made in the Tenth Census to obtain the number of the population on the 1st of June who are so sick or disabled as to be unable to pursue their ordinary avocations, and also to give the causes of such sickness or disability. This attempt has never been made before in this country, and in other countries has only been systematically made, as far as I am aware, in the three Censuses of Ireland for 1851, 1861, and 1871. It is too soon yet to speak of the results of this attempt, as the computations have not been made, but it is evident that either the returns will prove to be too imperfect to be of much, if any, statistical value, or the amount of sickness is much smaller than has been usually supposed. Taking the State of Rhode Island, where the Census was taken under the direction of a skilled superintendent, Dr. Snow—who had so small a territory to deal with that he could make sure of the knowledge and training of almost all his enumerators, and where it is to be presumed the population schedules have been filled out with the greatest accuracy and completeness—it is found by a count that the number reported sick and disabled, aside from those reported as blind, deaf and dumb, insane, crippled, etc., was a total of 3,276 out of a population of 276,528, being in the ratio of 11.8 per thousand. Comparing this with the results of the Irish Census of 1861, I find that the proportion of sick at their own homes reported in Ireland varied from 1 in 142 to 1 in 942 in the different localities specified, which is in the ratio of 7.04 and 1.06 per thousand respectively. It is usual to compute that for every case of death in a community there are two cases constantly sick.

It will be seen, if the estimate is correct, the Rhode Island Census comes much more near completeness than that of Ireland. The only other source of comparison which I have thus far found available is that of the health of the army, in which I find that among the white troops, for the year 1877, the average number constantly on the sick roll was 44 per thousand of mean strength.

Upon the whole, it seems probable that the results from the Rhode Island count are nearer the truth than any other data which we possess.

In conclusion, I think the Association will be glad to know that the work of tabulating these returns of deaths is going on rapidly, and that the results will be published in a form which will be found more satisfactory than the forms heretofore used, because it will present the comparison of mortality rates of counties and groups of counties, as well as of entire States. The importance of these mortality records is very great, in fact, because for very large portions of this country they give almost the only positive data we can get for comparison of the truthfulness of different localities; in fact, because they will serve both as a pattern and an incentive for scientific work in this direction on the part of municipalities and states.

REPORT ON CLINICAL AND METEOROLOGICAL RECORDS, BY DR. N. S. DAVIS.—This report, which was an able and exhaustive one, closed with the following recommendations:

1. That a committee of five be appointed by the President of this Association, to be called the "Standing Committee on Atmospheric Conditions, and their Relations to the Prevalence of Disease."

2. That said committee be authorized to select such representative places as will best indicate the atmospheric conditions in the more important climatic sanitary districts of the United States (to be not less than six nor more than twelve in number), and establish therein means for continuous observation and record of appreciable conditions of the atmosphere, according to the most approved methods, and of the origin and prevalence of all acute diseases.

The third resolution recommends, in determining the organic and other oxiding elements of the atmosphere, the use of the thallium paper, as prepared by Schone; and for determining the presence and relative quantity of ammonia and other organic products, the use of the pumice-stone method recommended by Professor Remsen, in his recent report to the National Board of Health.

4. The committee require a full statement of the results of the work done at each station, on the 1st of January and July of each year.

5. Appropriate $500 to carry out the purposes of these recommendations; a full statement of the amount received and expended to be reported at the next annual meeting.

The report concludes as follows:

In submitting the foregoing report, your committee are not unmindful of the magnitude of the work proposed, and of the difficulties that will be encountered in its prosecution. But if it is undertaken and prosecuted with persevering industry, much aid may be obtained from the co-operation of local medical societies and boards of health, and results of great value will be obtained, which, from the nature of the problems involved, cannot be obtained without such combined action of many working on parallel lines, under a uniform supervision. And should the foregoing recommendations meet your approval, it will inaugurate a change in medical society work by substituting, in part, at least, carefully devised plans for continuous investigation for adding to the stock of medical knowledge and establishing more complete methods, instead of depending wholly on individual and fragmentary contributions, and will thereby greatly enhance the value of social organizations in the profession.

The report is signed by Doctors N. S. Davis, J. M. Toner, and Henry O. Marcy. Doctors S. M. Bemis and W. H. Geddings, the other members of the committee, were absent, and did not sign the report.

The report was adopted and referred to the Committee on Publications.

The recommendations of the Committee were adopted.

